# Novel Strategies to Profile SARS‐CoV‐2 and Human Lung Proteome: Inflammatory Pathways in the Spotlight

**DOI:** 10.1155/bmri/5571277

**Published:** 2025-11-19

**Authors:** E. Mankayi, T. E. Chiliza, N. E. Mvubu

**Affiliations:** ^1^ School of Laboratory Medicine and Medical Sciences, College of Health Sciences, University of KwaZulu-Natal, Durban, South Africa, ukzn.ac.za; ^2^ School of Life Sciences, College of Agriculture, Engineering and Science, University of KwaZulu-Natal, Durban, South Africa, ukzn.ac.za; ^3^ Igagasi Biotech, TIA Platform, Durban, South Africa; ^4^ Department of Medical Biosciences, Faculty of Natural Sciences, University of the Western Cape, Cape Town, South Africa, uwc.ac.za

**Keywords:** cytokine storm, lateral flow immunoassays, lung proteome, phage display, SARS-CoV-2, single-cell RNA sequencing

## Abstract

Severe acute respiratory syndrome coronavirus 2 (SARS‐CoV‐2), the causative agent of COVID‐19, has caused widespread morbidity and mortality worldwide. SARS‐CoV‐2 infection triggers innate and adaptive immune responses, but excessive cytokine release can drive hyperinflammation, acute respiratory distress syndrome and poor clinical outcomes. Although serological and molecular assays, such as ELISA and RT‐qPCR, remain central to COVID‐19 diagnostics, they have limited capacity to reveal host–pathogen interactions at the tissue level. Therefore, profiling the human lung proteome offers a powerful strategy to identify molecular signatures associated with viral pathogenesis and disease severity. This review emphasises emerging technologies that advance lung proteome profiling during SARS‐CoV‐2 infection. Novel strategies include phage display for high‐throughput identification of antibody–antigen interactions, yeast two‐hybrid for mapping virus–host protein interactions and lateral flow immunoassays for rapid, point‐of‐care detection. Conversely, omics‐based technologies such as single‐cell RNA sequencing, microarrays and mass spectrometry are transforming our understanding of the lung proteome by revealing patterns of gene expression, protein abundance and immune heterogeneity. Therefore, comparing these conventional diagnostic assays with innovative approaches, we highlight their unique contributions to lung proteome research. These tools not only improve diagnostic precision but also hold the potential to uncover biomarkers for early risk stratification and therapeutic targeting. Prioritising integrative proteome‐focused strategies may ultimately guide personalised interventions and enhance preparedness for future viral outbreaks.

## 1. Introduction

Coronavirus disease (COVID‐19), an infection caused by a novel single‐stranded RNA virus, severe acute respiratory syndrome coronavirus 2 (SARS‐CoV‐2), typically affects the respiratory system and other primary organs of the body such as the heart, brain and kidneys [1]. This infectious disease has become a global health emergency as it has accounted for over a million fatalities ever since being declared a pandemic [2]. According to the World Health Organisation [3], over 775 million cases have been confirmed and over 7 million deaths have been reported to date globally. SARS‐CoV‐2 continues to spread and evolve, resulting in strains which have acquired a competitive advantage and therefore have been labelled as variants of concern (VOCs) [4]. Currently, WHO [5] is tracking various SARS‐CoV‐2 variants such as BA.2.86 and JN.1 labelled as the variants of interest (VOIs). Additionally, SARS‐CoV‐2 variants JN.1.7, JN.1.18, KP.2, KP.3 and LB.1 have exhibited increasing prevalence globally, hence labelled the variants under monitoring (VUMs). The success of the SARS‐CoV‐2 virus as a global pathogen is linked to the presence of several virulence factors, including three (3) of its proteins found on the surface envelope and one (1) found in the ribonucleoprotein core. These four (4) structural proteins have been said to be the primary targets of potential vaccines [6]. Vaccination has been implemented as one of the strategies for mitigating COVID‐19 [7]; however, due to the constantly mutating SARS‐CoV‐2 virus, the effectiveness of vaccines has varied, especially with the omicron variant [8]. Hence, it is highly significant to maintain the vaccination rate to control the spread of the virus [7].

Infection with the virus can cause manifestations ranging from mild to severe critical conditions, such as the acute distress syndrome (ARDS), pneumonia and even disseminated intravascular coagulation (DIC) due to activation of the immune system and high levels of cytokine production [9]. According to recent publications, SARS‐CoV‐2 infection is a key factor in the dysregulation of the immune response, resulting in the activation of inflammatory pathways that contribute to COVID‐19 severity [10]. Multiple studies have shown that hyperinflammatory responses lead to a cytokine storm, a life‐threatening clinical condition [11].

Pathogenesis of SARS‐CoV‐2 mainly involves viral entry, whereby the SARS‐CoV‐2 S protein binds to a membrane surface receptor, angiotensin‐converting enzyme 2 (ACE2), and is therefore initiated by the host cell transmembrane serine protease type 2 (TMPRSS2) mediating viral replication [1]. Despite various studies being conducted to date, the pathogenesis of this virus remains largely unknown due to its high rate of mutations [2]. In addition to vaccine development and administration currently being used to prevent SARS‐CoV‐2 infections, a better understanding of inflammatory pathogenesis pathways associated with the activation of cytokine signalling pathways is critical in developing novel therapeutic strategies for mitigating new infections [7].

Since the emergence of SARS‐CoV‐2, diagnosis has played a pivotal role in maintaining the spread of the virus [12]. The tools that have been used for SARS‐CoV‐2 diagnostics include serological and immunological‐based assays, omics tools and several other molecular tools. These tools collectively contribute to facilitating the understanding of SARS‐CoV‐2 pathogenesis as well as vaccine and drug development [13].

Despite extensive research on COVID‐19 pathogenesis, a significant gap remains in understanding how lung tissues specifically respond to SARS‐CoV‐2 infection. Diagnostics of COVID‐19 is crucial; hence, the development of rapid, reliable, specific and sensitive tools is pivotal. Previously, reviews focused on immune responses or diagnostics, while fewer have examined lung‐specific proteomic alterations; however, this review uniquely emphasises the strategies for profiling the lung proteome, where SARS‐CoV‐2 pathology is most pronounced. Additionally, it prioritises emerging technologies such as phage display, yeast two‐hybrid (Y2H) and single‐cell omics, which have the potential to reveal host–virus interactions, biomarker signatures and therapeutic targets. Focusing on these technologies will bridge the gap between general diagnostics and lung‐specific proteomic insights. The central objective of this review is to link the dysregulated immune pathways that are observed in COVID‐19 with the need for advanced diagnostic and proteomic strategies.

## 2. The Immune Response to SARS‐CoV‐2 Infection

During SARS‐CoV‐2 infection, the innate immune responses are initiated by multiple host pattern recognition receptors (PRRs), including toll‐like receptors (TLRs), intracellular retinoic acid‐inducible gene‐1 (RIG‐1)‐like receptors (RLRs) and melanoma differentiation‐associated protein 5 (MDA5). PRRs recognise the viral pathogen–associated molecular pattern (PAMP) and damage‐associated molecular patterns (DAMPs); thereafter, they activate inflammatory pathways which induce the production of proinflammatory cytokines such as interleukin (IL), interferon (IFN) and tumor necrosis factor (TNF) as well as chemokines [10], which recruit more innate immune cells including phagocytes (macrophages and neutrophils), monocytes, natural killer (NK) cells and dendritic cells (DCs) to the site of infection [2]. Furthermore, cells such as mast cells, basophils and innate lymphoid cells, which are part of the innate immune responses, are critical and contribute to degranulation and production of cytokines during SARS‐CoV‐2 infection [14]. Nevertheless, recent studies have indicated that SARS‐CoV‐2 has developed immune inhibitory mechanisms that hinder the antiviral immune responses. These strategies employ nonstructural proteins (NSP‐1 and NSP‐12) to inhibit RIG‐1‐independent innate immune responses and IFN production [15]. Furthermore, it has been established that the membrane (M) protein, a structural protein of SARS‐CoV‐2, inhibits the action of MDA5, thus contributing to the inhibition of the host antiviral response.

The adaptive immune system, which is the second line of defence, comprises two major cell types, T and B cells [16]. This line of defence plays a crucial role in viral infection control and the production of immune memory [17]. T cells mainly eliminate the cells of the body that have been infected by the virus, whereas B cell function in the production of antibodies, which attack invading pathogens [18]. T cells recognise antigens that have been encountered by the body and will either assist B cells in antibody production or eliminate the infected cell. These cells comprise CD8^+^ (cytotoxic) T cells, which bind to antigens to kill the infected cells and CD4^+^ (helper) T cells, which assist B cells in the production of antibodies that inhibit foreign pathogens and/or activate CD8^+^ T cells for proliferation [16]. Furthermore, when B cells encounter SARS‐CoV‐2 antigens, they produce specific antibodies such as IgM, IgA, IgG and neutralising antibodies that inhibit the mechanisms of SARS‐CoV‐2 antigens through immune response, in addition to preparing these antibodies for protection against future infections [19].

Cytokines are signalling proteins released by immune cells during infection with SARS‐CoV‐2 to control the activity of inflammation [20]. Production of these signalling proteins regulates host immune responses to defend the body during infection; however, once overproduced, they lead to what is known as a cytokine storm [21]. It has been established that proinflammatory cytokines such as IL‐1, IL‐6 and TNF‐*α* aggravate COVID‐19 progression, whereas anti‐inflammatory cytokines may lessen inflammation [22]. A study by Wolszczak‐Biedrzycka et al. [23], which analysed serum samples of 100 COVID‐19 patients in comparison with 50 healthy controls, confirmed the release of cytokines in COVID‐19 patients by revealing that COVID‐19 patients had elevated concentrations of some cytokines (IL‐1ra, IL‐2ra, IL‐6 and IL‐8), which can serve as potential SARS‐CoV‐2 biomarkers compared to asymptomatic patients. Another study by Cabaro et al. [24], which analysed cytokine profiles of COVID‐19 patients, revealed that infection with SARS‐CoV‐2 activates Type 1, Type 2 and Type 3 immunity, resulting in increased concentration levels of proinflammatory cytokines such as IL‐6.

Cytokine storm is an immune reaction to the excess release of cytokines into the body; this reaction has been referenced in various infectious diseases; hence, it is mostly associated with an uncontrollable inflammatory response [25]. Cytokine storm may have a positive response on some immune cells; however, more innate immune cells may be recruited, which may lead to organ damage. Previous literature on cytokine storm in COVID‐19 patients has shown that COVID‐19 patients have elevated levels of proinflammatory cytokines and chemokines. Chemokines are chemotactic cytokines that direct the movement of leukocytes to the site of inflammation [26].

A study by Zhang et al. [27], which investigated changes in the monocyte morphology and activation correlating to the severity of COVID‐19, outlined two different cytokine storm pathogenesis mechanisms. They stated that some COVID‐19 patients who were susceptible to cytokine storm had a genetic mutation in the proteins released by cytotoxic cells, which led to a positive feedback mechanism. However, those who had inflammatory responses to infection exhibited cytokine storm, leading to a negative feedback mechanism.

Additionally, recent studies have indicated that since the beginning of the COVID‐19 pandemic, ARDS has been associated with a high number of fatalities in patients infected with COVID‐19 [11]. Therefore, it can be said that the overproduction of active immune molecules (IFN, ILs, chemokines, colony‐stimulating factors and TNFs) results in a cytokine storm and the associated ARDS [21].

### 2.1. TLR Signalling Pathway

TLRs are protein molecules belonging to a family of innate immune receptors [28]. They are defined by an extracellular domain consisting of leucine‐rich repeat (LRR) motifs, a Type‐1 transmembrane domain and an intracellular domain, cytoplasmic Toll interleukin receptor 1 (TIR) [29]. Based on localisation, there are cell surface TLR molecules (TLR‐1, TLR‐2, TLR‐4, TLR‐5, TLR‐6 and TLR‐10) and endosomal TLR molecules (TLR‐3, TLR‐7, TLR‐8 and TLR‐9) [30].

TLRs recognise ligand molecules shared by pathogens and lead to the activation of the innate immune system. They drive two signalling pathways (MYD88‐dependent pathway and TRIF‐dependent pathway) which lead to the production and activation of proinflammatory cytokines, chemokines and Type‐1 IFNs [2]. the myeloid differentiation primary response 88 (MYD88)‐dependent pathway employed by almost all TLRs except TLR3, which signals through the TRIF‐dependent pathway, is initiated by the recruitment and activation of Toll interleukin 1 receptor adaptor protein (TIRAP), which in turn recruits and activates the MYD88 protein [31]. The MYD88 protein activates the Interleukin‐1 receptor‐associated kinase (IRAK‐1/4) complex, which in turn recruits the TNF receptor‐associated factor (TRAF‐6) molecule, allowing interaction with the TAB 6 protein to further activate the TGF‐Beta activated kinase (TAK‐1 protein) [32]. For downstream signalling, the IKK protein complex is activated, and the NF‐*κ*B protein associates with the I*κ*B*α* protein. However, due to the association with the I*κ*B*α* protein, the NF‐*κ*B protein is unable to enter the nucleus; therefore, the IKK protein phosphorylates the I*κ*B*α* protein, resulting in its degradation by proteasomes [33]. The NF‐*κ*B then enters the nucleus and drives the transcription of genes which produce proinflammatory cytokines and chemokines [2].

The TRIF‐dependent pathway, on the other hand, is initiated by the recruitment of the TRAM protein to the TIR domain [34]. TRIF then activates the RIP‐1 and TRAF‐3 proteins, which enable the TBK‐1 protein to activate the IRF‐3 protein complex [30]. Upon its activation, it enters the nucleus and drives transcription of Type‐1 IFN [35]. According to Yang et al. [36], COVID‐19 patients exhibited sufficient PAMP/DAMPs recognised by TLRs in their bloodstream and lungs. In their study, it is mentioned that the SARS‐CoV‐2 S protein binds to TLR‐1, TLR‐4 and TLR‐6, and based on their *in silico* analysis, TLR‐4 exhibited a strong interaction with the S protein; hence, the conclusion that the high binding affinity of TLR 4 is due to its involvement in COVID‐19 pathogenesis.

TLR4 recognises the lipopolysaccharide (LPS) from SARS‐CoV‐2 and becomes activated, expressing some inflammatory cytokines [36]. Due to this, clinical observations of COVID‐19 patients exhibited elevated proinflammatory cytokines, which ultimately led to a cytokine storm [37]. Research has also shown that activation of TLR 4 mediates sepsis following SARS‐CoV‐2 infection [38]. Another study by Zheng et al. [39] confirmed that SARS‐CoV‐2 E protein binds TLR 2 and activates its inflammatory pathway. Planes et al. [40] further characterised the E protein and found that it activates the NF‐*κ*B factor, driving the production of CXCL8, an inflammatory chemokine. Activation of TLR 2 during the pathogenesis of COVID‐19 is critical; hence, screening of COVID‐19 patients is highly significant in the prevention of disease progression.

### 2.2. Janus Kinase (JAK)/STAT Signalling Pathway

The JAK‐STAT pathway is a signalling mechanism for signal transduction of multiple growth factors (cytokines) from the extracellular region into the nucleus of the cell [41]. The extracellular factors control the expression of genes that are widely involved in cell proliferation, immunity and cell differentiation [42]. This pathway consists of two components (JAK and STAT), which both play significant roles in the cell. JAK is an intracellular tyrosine kinase that consists of JAK1, JAK2, JAK3 and TYK2. It is involved in the transduction of cytokine‐mediated signals from the extracellular region to activate the functioning of the signal transducers and activators of transcription (STATs) [2]. The STAT protein is an intracellular transcription factor that consists of STAT1, STAT2, STAT3, STAT4, STAT5a, STAT5b and STAT6 [43]. This protein contains different domains with diverse functions in the JAK/STAT pathway, mainly the N‐terminal domain, which aids in dimer formation; the coiled coil domain, which acts as a dimerisation tag; the DNA binding domain, which assists the STAT in binding to the target DNA; the SH2 domain, which mediates docking of the phosphorylated tyrosine and lastly, the C‐terminal domain involved in gene expression [44].

The extracellular region of the cell has cytokine receptors which receive signals in the form of cytokines (IFN, IL and other growth factors) and therefore bind to them [41]. Once these receptors interact with these signals, the JAK proteins associated with the receptors are brought into proximity, resulting in conformational changes [45]. The receptor‐associated JAK proteins phosphorylate, creating a docking site for the STAT protein, which docks to the phosphotyrosine site through its SH2 domain. Continuing with phosphorylation, the STAT protein deattaches from the receptor, mediating STAT dimerisation, which translocates the signals to the nucleus, regulating gene expression [46].

Recent studies have shown that cytokine storm due to SARS‐CoV‐2 infection has elevated the activation of the JAK/STAT pathway, causing COVID‐19 patients to have higher levels of inflammatory cytokines (such as IL‐2, IL‐4 and IL‐6), which regulate inflammation and therefore recruit innate immune cells, including NK cells, macrophages and chemokines [47]. According to Satarker et al. [48], the necessity of cytokines to fight SARS‐CoV‐2 infection is very much evident; however, excessive inflammation due to cytokine storm leads to immunopathological impairments of COVID‐19, which progress to critical conditions such as ARDS.

A study conducted by Aliyu et al. [26] outlined that the JAK/STAT pathway is also activated by some proinflammatory cytokines (IL‐6, IFN‐*γ*, AgII/AT1R and GM‐CSF), which leads to overproduction of cytokines, causing excessive inflammation and cytokine storm. IL‐6 secreted by B cells, T cells, macrophages and other cell types is said to have increased levels in COVID‐19 patients. It is associated with hyperactivation of the immune cells, leading to cytokine storm [2]. This IL activates the JAK/STAT pathway in a two‐way series (classical signalling and trans‐signalling). IL‐6 binds to its receptor (mIL‐6R in classical signalling and sIL‐6R in trans signalling), interacts with the gp130 protein, forming complexes that activate JAK/STAT3 signalling [11]. According to Yang et al. [49], IL‐6 is a significant biomarker of COVID‐19; therefore, targeting its receptor signalling can be a promising immunotherapeutic strategy.

IFNs are categorised into three (3) different types (IFN I, IFN II and IFN III) which are secreted by various cell types (such as leukocytes, T cells and NK cells). Based on recent studies, COVID‐19 patients expressed elevated levels of IFN‐*γ*, a type II IFN, and reasonably lower levels of IFN I and III [2]. Yang et al. [11] stated that during SARS‐CoV‐2 infection, IFN I is considered the first line of protective response, as it fights the viral infection to promote viral clearance and immune responses. Furthermore, the JAK1/TYK2/STAT1/2 pathway is activated, and to find promising defense mechanisms, more studies are needed on how the IFN function can be inhibited.

During SARS‐CoV‐2 infection, angiotensin II (Ang II) interacts with the receptor angiotensin II type 1 (AT1) to activate the JAK/STAT pathway. Additionally, GM‐CSF activates the JAK/STAT pathway by binding to a subunit of the GM‐CSF receptor [41]. These interactions regulate excessive cytokine (such as DCs) production. However, the GM‐CSF function can be inhibited by the JAK2 inhibitor; hence, the JAK2 inhibitor can be a potential key factor in cytokine storm management in COVID‐19 [25].

### 2.3. NF‐*κ*B Pathway

NF‐*κ*B is a transcriptional factor that gets activated during inflammatory responses. It consists of various proteins, namely, Rel‐like structural domains p65 (RelA), RelB, c‐Rel, p105/p50 (NF‐*κ*B1) and lastly p100/p52 (NF‐*κ*B2), which form complexes as they bind together. This transcriptional factor plays a key role in immune responses, cell proliferation, differentiation and apoptosis [41]. The NF‐*κ*B pathway is initiated by TNF‐*α*, a factor that is capable of driving apoptosis. Additionally, it can drive cell survival pathways through the production of cytokines in the NF‐*κ*B pathway, as it inhibits apoptosis [25]. Basically, TNF‐*α* has 2 mechanisms, which may either lead to cell death or cell survival. In the apoptosis mechanism, the TRADD recruits the receptor‐interacting protein (RIP) together with FADD, central regulatory molecules, which initiate the caspase cascade, inducing cell death, whereas in the NF‐*κ*B pathway, TRADD recruits RIP and TRAF2 for NF‐*κ*B activation, leading to cell survival [2].

The cell consists of a TNF receptor (TNFR‐1), which is located on the plasma membrane. This receptor has a death domain (DD) on the intracellular side that is kept in an inactive state by the silence of death domain (SODD) [50]. The binding of the TNF‐*α* molecule to its receptor causes conformational changes on the receptor, resulting in the release of SODD from the DD. The DD, therefore, interacts with the TRADD protein, which recruits the TRAF2 protein, forming a TRADD‐TRAF complex that recruits the IAP protein [51]. The IAP protein binds with TRAF2, inhibiting apoptosis; this, however, leans towards the NF‐*κ*B pathway. TRAF2 then recruits the RIP protein, whose function is to activate the IKK molecule for kinase activity [52]. On the other hand, the NF‐*κ*B molecule is in an inactive state as it is attached to the I*κ*B*α* protein that disguises its NLS signalling, inhibiting translocation to the nucleus; however, for activation, the I*κ*B*α* must be removed [53]. To remove the I*κ*B*α* molecule, the IKK molecule phosphorylates the I*κ*B*α* and will therefore be degraded by proteasomes, detaching from the NF‐*κ*B molecule, promoting its translocation to the nucleus to activate transcription of cell proliferation [54].

During infection with SARS‐CoV‐2, various viral proteins promote the hyperactivation of the NF‐*κ*B pathway [2]. A study by Hariharan et al. [55] revealed that the production of cytokine storm and severity of COVID‐19 can be reduced by immunomodulation during NF‐*κ*B activation; additionally, degradation of I*κ*B and TNF‐*α* inhibition may also play significant roles in the reduction of cytokine storm production. Another study by Kircheis et al. [9], which investigated therapeutic approaches which aim at inhibiting the whole cascade of proinflammatory cytokines and chemokines, revealed that some proteasome inhibitors (carfilzomib or ixazomib) are regarded as powerful biomarkers for NF‐*κ*B inhibition, downregulating cytokine storm in COVID‐19 patients.

## 3. Tools Exploited to Profile Human Lung Proteome During SARS‐CoV‐2 Infection

As an emerging pathogen, SARS‐CoV‐2 has highlighted the significance of timely and precise diagnosis. Diagnostic tools became crucial during the COVID‐19 pandemic; therefore, the development of techniques such as serological and molecular tools (Table 1) that can be exploited to profile the human lung during SARS‐CoV‐2 infection will remain vitally significant as they assist in the understanding and control of SARS‐CoV‐2’s widespread and other future outbreaks. The use of novel strategies such as phage display and lateral flow immunoassay (LFIA) can, however, address this gap.

**Table 1:** Summary of novel strategies used to profile the human lung proteome during SARS‐CoV‐2 infection.

**Technique**	**Principle**	**Strength**	**Limitation**	**Application to lung proteome profiling**	**Novel contribution to COVID-19**
ELISA	Antigen‐antibody binding with enzymatic detection [56].	High sensitivity, cost‐effective and scalable [57].	Limited multiplexing, requires lab infrastructure [58].	Detection of antibodies in patient sera [59].	Identification of antibody responses to SARS‐CoV‐2 proteins [60].
IFA	Antigen–antibody detection with fluorescence visualisation [61]	High specificity [62], localisation of viral proteins [63].	Requires fluorescence microscopy [62], low throughput [64]	Localisation of viral proteins in lung tissue [63].	Mapping SARS‐CoV‐2 protein distribution within host cells [65].
RT‐qPCR	Amplification of viral RNA via reverse transcription [66]	Gold standard, early detection and variant tracking [61].	False negatives, time consuming and costly [67].	Detection of viral RNA in lung samples [68].	Rapid identification of infection and viral load [69].
CRISPR‐based assay	Guide RNA and Cas enzyme cleavage with reporter signal [70].	Rapid, highly specific and low false negatives [67].	Still developmental and requires expertise [67].	Detection of viral RNA in respiratory tissues [70].	Next‐gen diagnostics with point‐of‐care potential [70].
scRNA‐seq	Sequencing of transcripts at single‐cell resolution [71].	Reveals cell heterogeneity and unbiased profiling [72].	Limited by tissue availability and costly [73].	Mapping immune and epithelial cell responses in lungs [74].	Uncovered host‐pathogen dynamics in lung tissue [75].
Microarrays	Hybridisation of fluorescently labelled cDNA to gene chips [76].	High‐throughput and simultaneous gene detection [77].	Less sensitive than sequencing and dependent on probe design [77].	Profiling gene expression patterns in lung tissue [78].	Identified antibody and gene expression signatures in COVID‐19 [78].
Phage display	Display of peptides/antibodies on phage coat proteins [79].	High‐throughput, epitope mapping and library generation [80].	Requires complex library design [81].	Identification of antibody repertoires against lung proteome targets [82].	Discovery of neutralizing antibodies and vaccine epitopes [81].
Yeast two‐hybrid	Fusion proteins tested for interaction in yeast [83].	Effective for mapping protein–protein interactions [83]	False positives and limited to binary interactions [84]	Identification of SARS‐CoV‐2‐host protein interactions [84].	Revealed more than 300 novel proteins interacting with viral proteins [84].
LFIA	Antigen–antibody capture on nitrocellulose strips [85].	Point‐of‐care [86], rapid and low cost [87].	Lower sensitivity [87] and limited quantification [88].	Detection of viral antigens or antibodies in patient samples [89].	Accessible COVID‐19 screening in low resource settings [87].

### 3.1. Serological Assays

**Figure 1:**
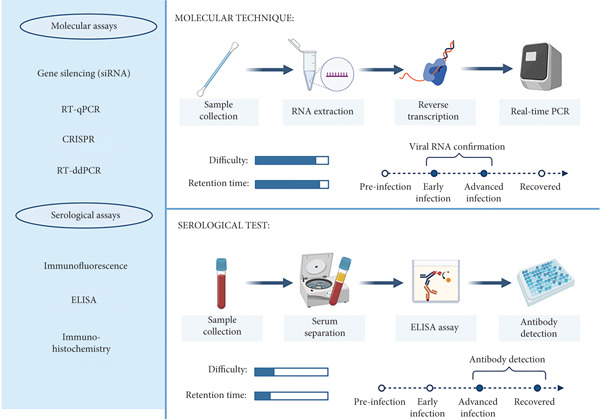
SARS‐CoV‐2 molecular and serological techniques. SARS‐CoV‐2 RNA detection from a nasopharyngeal/oropharyngeal swab through multiple steps, including RNA extraction, reverse transcription, and RT‐qPCR. Upon detection, the retention time of results is produced within 24 h (top). Antibody detection from serum or plasma samples in response to viral infection. Upon sample collection, SARS‐CoV‐2 antigen and anti‐SARS‐CoV‐2 antibody are coated for interaction and incubated with a retention time of 30 days after onset (bottom). The image was created in http://biorender.com/.

Serological assays are subsets of immunological tests whose function is to detect and quantify pathogen‐specific antibodies (IgG, IgA and IgM) in a blood sample [90]. These assays have been employed to provide insight into the changes that may occur in the human lung proteome during infection with SARS‐CoV‐2 and, therefore, profile biomarkers for potential therapeutic targets of COVID‐19 [91]. These assays include, but are not limited to, immunofluorescence assay (IFA) and enzyme‐linked immunosorbent assay (ELISA) [92], immunohistochemistry [93] and peptide microarrays [94] (Figure 1). Current literature has shown that the production levels of the detected immunoglobulins differ at different stages of COVID‐19 infection. Yu et al. [95] established that there were higher levels of IgG and IgM in severe COVID‐19 patients, whereas the study [96] exhibited higher levels of IgA during the early stage of COVID‐19 infection.

An IFA uses antibodies and fluorescence for visualisation of the fluorescence marker [61]. During this assay, antibodies bind to the protein of interest, allowing their conjugation with fluorescence for the detection of the protein of interest with fluorescence microscopy. IFA is a very sensitive and specific tool [62], such that it can detect lower levels of antigens exhibiting high specificity. These antigens are visualised through fluorescence microscopy [61]. According to Michel et al. [64], IFA can be applied in anti‐SARS‐CoV‐2 antibody detection by spotting harvested SARS‐CoV‐2‐infected cells together with serum samples onto a microscope glass slide, washing with their conjugates and therefore, observing under fluorescence microscopy. A study by Zhang et al. [63], which focused on the localisation of SARS‐CoV‐2 proteins in host cells (HEp‐2 and Caco‐2 cells) using the IFA assay, demonstrated that some SARS‐CoV‐2 proteins (M, ORF6, ORF7a and NSP15) were related to the Golgi apparatus. They further mentioned that most of the SARS‐CoV‐2 proteins are related to numerous cell sites; hence, the necessity for further investigation on viral protein localisation as it may provide a better understanding in the fight against SARS‐CoV‐2 infection. Another study by Edouard et al. [65], where they applied IFA to detect anti‐SARS‐CoV‐2 antibodies and further compare its performance with the ELISA anti‐SARS‐CoV‐2 IgG kit, found that the assay specificity was greater than 80% for the antibodies IgA, IgM and IgG. They further mentioned that IFA reached a significant consensus of 86% with the ELISA anti‐SARS‐CoV‐2 IgG kit, making it a potential marker for association with COVID‐19 severity. To profile the human lung development, Clair, Bramer [97] stained human lung tissue samples with various primary antibodies and found that as the lung develops, cellular homeostasis is very crucial due to the modulation of proteins. Their study, therefore, suggests a global shift towards maintaining stable conditions in the event of respiratory impairments due to SARS‐CoV‐2 infection and other infectious diseases.

ELISA is a laboratory technique that is widely used for the detection and quantification of antibodies and/or antigens related to SARS‐CoV‐2 [56]. This tool uses biological samples from patients infected with COVID‐19 for incubation with either antibody or antigen for binding [98]. During this assay, the anti‐SARS‐CoV‐2 antibody, SARS‐CoV‐2 antigen and enzyme‐labelled antibody are coated on the surface of a well plate, washed, and an enzyme substrate is added to produce a calorimetric change; thereafter, detection is done using an ELISA reader to measure the level of intensity [58]. Its application in SARS‐CoV‐2 is due to the production of high levels of sensitivity and specificity and its ability to process several samples [57]. A study by Luo et al. [99], whose focus was detecting SARS‐CoV‐2‐specific antibodies using the ELISA assay, confirmed the production of IgG antibodies that acted against the N protein of SARS‐CoV‐2 through the assay’s high sensitivity and specificity and the use of multiple (120 sera and 1500 control samples) samples. Osterman et al. [100] investigated four automated antigen tests, including SARS‐CoV‐2 Ag ELISA, in comparison to a point‐of‐care testing rapid Ag test (SARS‐CoV‐2 Rapid Antigen Test) and found that the ELISA assay stayed above the required limit of at least 97% for specificity; however, it was lower than that of the rapid antigen test. Therefore, they concluded that the ELISA assay can detect SARS‐CoV‐2 N antigen of VOCs alpha and beta. Another study by Liu et al. [59] developed a fluorescence‐linked immunosorbent assay (ELISA‐based) using p‐Si‐MRh–labelled fluorescent IgG to detect SARS‐CoV‐2 spike S1 protein–specific IgG. Their method successfully detected the antibodies, and they concluded that ELISA‐based approaches could serve as an emerging toolbox for antibody labelling. Lastly, Gong et al. [101] profiled lung tissues and blood in ARDS. They evaluated lung injury by obtaining bronchoalveolar lavage fluid (BALF) and measuring protein concentrations using the ELISA assay. Following the LPS challenge, BALF protein levels were significantly higher than in the healthy control group, indicating increased lung injury and inflammation.

### 3.2. Molecular Techniques

Molecular tools consist of techniques that detect viral genes and are deemed specific, sensitive, simple and cost‐effective [12]. These techniques include, but are not limited to, real‐time reverse transcriptase polymerase chain reaction (RT‐qPCR), clustered regularly interspaced short palindromic repeats (CRISPR) and gene silencing through siRNA [60] (Figure 1).

RT‐qPCR is considered a gold standard for SARS‐CoV‐2 detection due to its advantageous properties, such as high sensitivity and specificity, early detection of SARS‐CoV‐2 and tracking of SARS‐CoV‐2 variants [61]. This technique requires a nasal or throat swab for RNA extraction [102], which is reverse transcribed into cDNA that is further amplified and detected through fluorescence dyes or probes [66]. In spite of the fact that RT‐qPCR is considered a gold standard detection technique, it has its limitations, such as being time‐consuming, yielding false negative results, and being costly [67]. A study by Dewald et al. [103], where they implemented a Lolli method approach for sample collection in children for SARS‐CoV‐2 detection and further analysis with RT‐qPCR, successfully detected approximately 80% of infected individuals with a sensitivity of 50% and specificity of 100% in healthy individuals. During their study, a total of 1,110,033 RT‐qPCR (698 day‐care facilities) was carried out with a retention time for 96.2% of the samples in less than 24 h. Additionally, when they monitored SARS‐CoV‐2 infection in schools, 65% of RT‐qPCR tests were positive. They concluded that for control of SARS‐CoV‐2 infection, sample collection through the Lolli method and further analysis with RT‐qPCR can become a powerful tool for surveillance of SARS‐CoV‐2 in schools and day‐care centers. Another study by Thieulent et al. [104], which was aimed at detecting SARS‐CoV‐2 and common canine enteric coronavirus simultaneously, developed and validated a multiplex one‐step RT‐qPCR assay and found that 34.5% of tested samples were positive for either virus, with SARS‐CoV‐2 being the most detected among the viruses under surveillance. They further mentioned that an increase in sample size and the versatility of the viruses in question may improve the experiment in the future. Recently, Ding et al. [69] compared the performance of RT‐qPCR with RT‐ddPCR and found that RT‐ddPCR outperformed RT‐qPCR in SARS‐CoV‐2 detection. This can, therefore, suggest the implementation of RT‐ddPCR in the future as it has demonstrated being a promising tool for the detection of SARS‐CoV‐2 and future outbreaks. Lastly, Bo et al. [68] explored the molecular mechanisms underlying silicosis; their study investigated differentially expressed proteins and genes (DEPs and DEGs) in lungs exposed to silica particles using omics tools and further confirmed the obtained results through RT‐qPCR. Through a microarray analysis, they found that 1769 DEGs were differentially expressed, with 952 DEGs upregulated and 817 DEGs downregulated and thereafter, expression patterns were confirmed to be consistent using RT‐qPCR.

CRISPR has emerged as one of the powerful tools for SARS‐CoV‐2 detection alongside RT‐qPCR [105]. According to literature, this tool provides results in minimal time, is sensitive to low levels of SARS‐CoV‐2 RNA and is highly specific, minimising false negative results. Despite these advantages, CRISPR is still in its development phase and therefore requires expertise for further practice [67]. During SARS‐CoV‐2 detection with the CRISPR system, gRNA binds to its specific SARS‐CoV‐2 RNA sequences, and samples are prepared for RNA extraction. RNA is then reverse transcribed into cDNA, allowing binding with gRNA, further activating the Cas enzyme for cleavage with reporter molecules, which produce a signal for detection [70].

### 3.3. Omics Tools

Omics tools are high‐throughput techniques with subsets that focus on specific categories to provide fundamental knowledge in the complex interactions surrounding biological systems [106]. Omics tools have played a crucial role in providing insight and understanding in the biology of SARS‐CoV‐2, host responses and potential diagnostic strategies [107].

**Figure 2:**
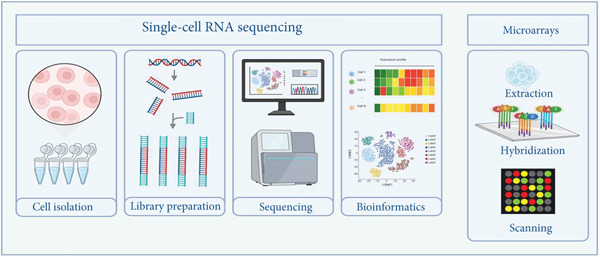
Omics tools exploited to profile the human lung during SARS‐CoV‐2 infection. Single‐cell RNA‐sequencing provides insight into cellular heterogeneity through gene expression at the single‐cell level. Upon reverse transcription, RNA is extracted from individually isolated cells, and a cDNA library is prepared. The library is sequenced using high‐throughput technologies and further analysed to quantify gene expression. Microarrays analyse gene expression and other genomic characteristics by labelling reverse transcribed RNA extracted from biological samples, applying the labelled cDNA to a microarray, and lastly, scanning and analysing the generated data for gene expression quantification. The image was created in http://biorender.com/.

Omics strategies integrate genomics, a biological study of the whole genome of an organism, to interpret genetic information. This tool comprises categories that include genome sequencing, genome annotation and epigenomics, combined with the exploitation of *in silico* strategies for the characterisation of the SARS‐CoV‐2 genome [108]. Additionally, the omics tool integrates transcriptomics that are widely used for gene regulation and expression through RNA‐seq, microarray analysis, single‐cell RNA sequencing (scRNA‐seq) and RT‐qPCR [109] (Figure 2). Lastly, proteomics is used for protein analysis through protein identification and protein separation strategies [110] while metabolomics characterises small molecules through metabolite profiling and mass spectrometry [111].

The scRNA‐seq is a very powerful technique with an approach of analysing and profiling gene expression, heterogeneity, and RNA transcripts of single cells, such as the lungs during SARS‐CoV‐2 infection. This technique involved cell isolation from lung tissues of COVID‐19 patients, library preparation, sequencing, and analysis through bioinformatics. scRNA sequencing has since been employed for its application during SARS‐CoV‐2 infection [71]. In a study by Speranza et al. [112] investigating the dynamics of SARS‐CoV‐2 infection in African green monkey lungs, it was revealed that gRNA remains highly stable following infection with irradiated SARS‐CoV‐2. They further investigated this claim through scRNA‐seq using cells isolated from the lungs and found that SARS‐CoV‐2 replicates primarily in pneumocytes, with macrophages steering the inflammatory response. This led them to conclude that scRNA‐seq can address the replication of SARS‐CoV‐2, dynamic responses from the host and gene expression. A study by Liu et al. [73], which addressed some limitations and other perspectives for exploiting scRNA‐seq, mentioned that understanding the pathophysiology of COVID‐19 in the human body is remarkable; however, it is limited to tissue sampling time, as most studies utilise samples collected from severe COVID‐19 patients. Thereafter, they mentioned a few single‐cell technologies that have been developed for profiling gene regulation, such as single‐cell assay for transposase accessible chromatin sequencing (scATAC‐seq) for immune‐related gene expression regulation, CITE‐seq for measuring receptor expression during SARS‐CoV‐2 entry and spatial transcriptomics. Another study by Travaglini et al. [74], which was aimed at developing a detailed molecular cell atlas of the adult human lung using this omics tool, was able to identify 14 novel populations across four different cell types (epithelial, endothelial, stromal and myeloid cells), further analysed their localisation, biochemical functions and interactions. Ronnberg et al. [72], a recent study which focused on investigating the heterogeneity of human lung mast cells through scRNA‐seq, was successful in their analysis and showed that multiple distinct genes were highly expressed as classical mast cell markers. Lastly, a study by Yoon et al. [75], which investigated the transcriptomic profiles of peripheral blood monoclonal cells (PBMCs) using scRNA‐seq after SARS‐CoV‐2‐infected and uninfected individuals developed pulmonary post‐acute sequelae of SARS‐CoV‐2 (PPASC) successfully revealed that myeloid lineage immune cells are altered in PPASC. This claim, therefore, allowed them to conclude that disruptions within the myeloid lineage immune cells are linked to the ongoing profibrotic mechanisms aiding the PPASC development.

Microarray technology is among the latest advanced diagnostic tools for the study of gene expression. This technique allows the detection of several genes simultaneously through labelling of cDNA with fluorescent dye, hybridising the labelled cDNA onto a microarray chip, and thereafter, scanning and analysing the fluorescent intensity, which indicates gene expression [76]. A study by Celikgil et al. [78], which was aimed at characterising the response of antibodies during infection with SARS‐CoV‐2, was successful through the development of a protein microarray and found that SARS‐CoV‐2 structural proteins showed different IgG levels in COVID‐19 patients. They further mentioned that the microarray technique is reliable for analysing COVID‐19 patient antibodies to neutralise SARS‐CoV‐2 variants. Another study by Damin et al. [77], which used a CovidArray technique to detect SARS‐CoV‐2 in nasopharyngeal swabs, was successful in detecting SARS‐CoV‐2 markers in a single assay. This microarray‐based assay demonstrated high specificity, sensitivity and minimal retention time; however, since it is still in its early stages, improvements such as detection using microtiter plates equipped with microplate fluorescence and an increase in sample size can be implemented in future studies.

### 3.4. Other Novel Strategies

**Figure 3:**
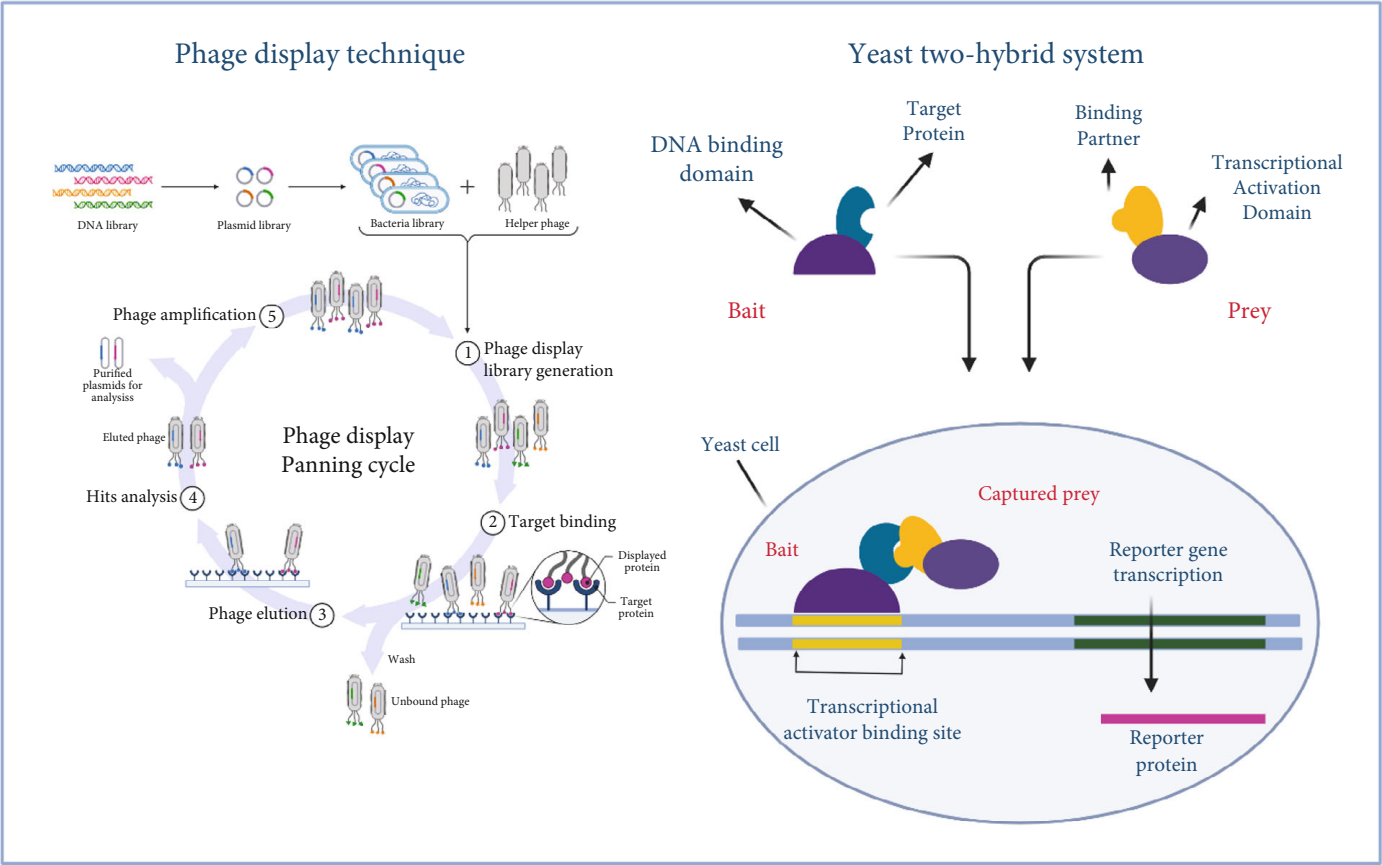
Novel strategies used to exploit the human lung during SARS‐CoV‐2 infection. The phage display technique exploits bacteriophages to display proteins on their surfaces. A diverse DNA library encoding numerous proteins is cloned into a phage genome. The resulting phage library is panned against a target protein, subjected to various rounds of washing, and the bound phages are eluted. Bound phages are amplified and further analysed by sequencing. The yeast two‐hybrid system utilises two proteins (bait and prey) and fuses them at their DNA‐binding domain and transcriptional activation domain. The plasmids are then introduced into a yeast cell, resulting in the expression of fusion proteins forming a transcriptional factor, which further activates the transcription of a reporter gene. The image was created in http://BioRender.com.

SARS‐CoV‐2 genome continues to evolve to better evade, adapt and replicate in the human immune system [113]. Multiple tools have been explored to profile the human lung proteome during SARS‐CoV‐2 infection, and to this day, only RT‐qPCR has been regarded as a gold standard for SARS‐CoV‐2 detection [114]. According to recent literature, novel strategies such as whole‐genome sequencing [115], phage display technology [79], Y2H, single‐cell proteomics [83] and LFIA [92] could enhance our understanding of the lung proteome and therefore contribute to the development of drug discovery (Figure 3).

Phage display technology has emerged as a very powerful technique for the study of protein interactions and the development of diagnostic targets for SARS‐CoV‐2. This technique exploits genetic diversity to display various mutant repertoires on the outer surface of a phagemid [79]. The phage display technique exploits bacteriophages by introducing foreign peptides/antibody fragments into a specific location; in turn, the bacteriophage displays foreign genes, which encode the coat protein on its surface [82]. According to Anand et al. [80], the phage display molecular tool can be applied in various aspects, such as monoclonal antibody selection, bioactive peptide, epitope mapping and drug delivery system development. They further mentioned its advantages, which included high‐throughput screening, improved efficiency and production of large‐sized phage display libraries. A study by Zhang [81] outlined that antibody repertoires are cloned into a phagemid, fused into pIII and therefore transformed into *Escherichia coli* cells. Furthermore, transformed cells are infected with a helper phage, generating a phage display library. Lastly, the library is panned against an immobilised antigen, followed by washing to remove unbound phages. Antigen‐specific phages are then eluted, propagated in *E. coli* and subjected to sequencing. This principle is therefore applied in the study of COVID‐19 vaccine development.

Y2H is a molecular technique used in the study of protein–protein interactions. This technique involves a protein of interest fused to a DNA‐binding domain, a protein assumed to interact with the protein of interest, and a yeast strain with a reporter gene. Here, a plasmid is constructed and transformed into a yeast strain. The transformants are then screened for protein‐protein interaction [83]. A study by Zhou et al. [84], which investigated the SARS‐CoV‐2–human protein–protein interactome using Y2H and tandem mass tag affinity purification–mass spectrometry (TMT‐AP‐MS) approaches, identified approximately 361 novel host proteins associated with SARS‐CoV‐2. The authors further noted that Y2H and TMT‐AP‐MS remain the most extensively used techniques for mapping protein–protein interaction networks, emphasizing the need for continued exploration using these approaches. These findings enhance our understanding of COVID‐19 pathobiology and support future drug development efforts.

LFIA, a widely used point‐of‐care technique, is employed for screening, diagnosis and monitoring of numerous infectious diseases [86]. During the COVID‐19 outbreak, LFIA was employed to detect antibodies against SARS‐CoV‐2 due to its ability to produce results in minimal time and is considered a cost‐effective and sensitive tool that can be advantageous for middle‐ and low‐income countries [87]. According to Li et al. [85], LFIA can be categorised into direct and competitive detection methods. Direct detection involves the detection of antibodies in the early stages of SARS‐CoV‐2 diagnosis, whereas competitive detection involves the detection of small molecules [86]. LFIA is not a widely studied technique in COVID‐19 diagnosis; however, based on a study by He et al. [89], some literature has shown interest in the application of LFIA on some SARS‐CoV‐2 proteins (N and S) as antigen targets. Consequently, this assay has become an important tool for SARS‐CoV‐2 detection, despite its limitation in distinguishing the efficacy of neutralizing antibodies against different variants [88].

## 4. Conclusion and Future Recommendations

COVID‐19 remains a global health challenge, with ongoing difficulties in developing effective therapeutic strategies. SARS‐CoV‐2 employs multiple mechanisms to disrupt key molecular pathways within both the innate and adaptive immune responses. As several studies have reported genomic similarities between SARS‐CoV‐2 and SARS‐CoV, it is plausible that SARS‐CoV‐2 employs comparable mechanisms to invade host cells and disrupt immune responses. It is also crucial to consider that factors such as mutation rate and evolving VOCs may bring about challenges for the treatment and prevention of COVID‐19. A fundamental understanding of the induced signalling pathways for SARS‐CoV‐2 treatment using newly developed drugs and even repurposed therapeutics is needed; hence, detection and prevention strategies for health precautions need to be employed. Several tools have been exploited to profile the human lung proteome during SARS‐CoV‐2 infection. However, there is still a gap in this regard as research areas base their focus on omics tools such as scRNA‐seq, whole genome sequencing and bronchoalveolar lavage only. Based on recent literature, emerging tools such as CRISPR, RT‐ddPCR, sandwich immunoassay, gene silencing through siRNA, and machine learning and AI can be used to profile the human lung proteome during SARS‐CoV‐2. LFIA, on the other hand, has primarily been used for diagnostic purposes; however, research areas have shown interest in its application to proteomics, more specifically in profiling the human lung proteome. Additionally, using Y2H and AT‐MS simultaneously may be more advantageous than exploiting each tool alone. Lastly, more and more novel studies are now exploiting the phage display technique for various infectious diseases to develop new vaccine targets; therefore, it can be suggested as one of the golden standards in profiling the human lung proteome during infection with SARS‐CoV‐2.

This review has highlighted a unifying theme, which is the urgent need for novel strategies to profile the lung proteome in SARS‐CoV‐2 infection. Bringing forward immune signalling pathways within the context of advanced diagnostic and proteomic technologies, we further highlight how these approaches can uncover biomarkers before disease severity and therefore, guide host‐directed therapy. The emerging strategies will broaden our ability to investigate lung‐specific host–virus interactions.

## Ethics Statement

This review did not involve the collection of new data or the conduct of experiments on humans or animals; hence, ethical approval was not applicable.

## Disclosure

All authors have read and agreed to the published version of the manuscript.

## Conflicts of Interest

The authors declare no conflicts of interest.

## Author Contributions

Conceptualisation: N.E.M.; investigation: N.E.M. and E.M.; data curation and analysis: E.M.; writing—original draft preparation: E.M.; writing—review and editing: E.M and N.E.M.; supervision: N.E.M. and T.E.C.; and project administration: N.E.M. and T.E.C.

## Funding

This study was supported by the South African Medical Research Council.

## Data Availability

Data sharing is not applicable to this article as no datasets were generated or analysed during the current study.
